# Supplementary material for: Experimental adaptation of dengue virus 1 to Aedes albopictus mosquitoes by in vivo selection
Source: Sci Rep. 2020 Oct 27;10:18404. doi: 10.1038/s41598-020-75042-4 (PMC7591890; doi:10.1038/s41598-020-75042-4)
Supplement: Supplementary file 1 — Supplementary Information. [file 41598_2020_75042_MOESM1_ESM.docx]

**Supporting information**

**Experimental adaptation of dengue virus 1 to *Aedes albopictus* mosquitoes by *in vivo* selection**

Rachel Bellone^1,2,#^, Sebastian Lequime,^2,3,4,#^ Henri Jupille,^1,#^ Giel P. Göertz,^5^ Fabien Aubry,^3^ Laurence Mousson,^1^ Géraldine Piorkowski,^6,7^ Pei-Shi Yen,^1^ Gaelle Gabiane,^1^ Marie Vazeille,^1^ Anavaj Sakuntabhai,^8^ Gorben P. Pijlman,^5^ Xavier de Lamballerie,^6,7^ Louis Lambrechts,^3,&^ Anna-Bella Failloux^1,&,*^

**Supplementary Fig. S1. Viral titers of cell culture supernatants used to run passages in the experimental selection for DENV-1 adaptation to *Ae. albopictus*.** Saliva were collected from 15-25 mosquitoes 19-21 days after infection and pooled to inoculate a monolayer of C6/36 *Ae. albopictus* cells. After 8 days at 28°C, cell culture supernatants were collected and provided to mosquitoes to run the next passage. Ten passages were performed. The supernatants were titrated by focus fluorescent assay on *Ae. albopictus* C6/36 cells. Viral titer was expressed in FFU/mL. Two biological replicates were performed for each viral strain, DENV-1 30A and DENV-1 1806.


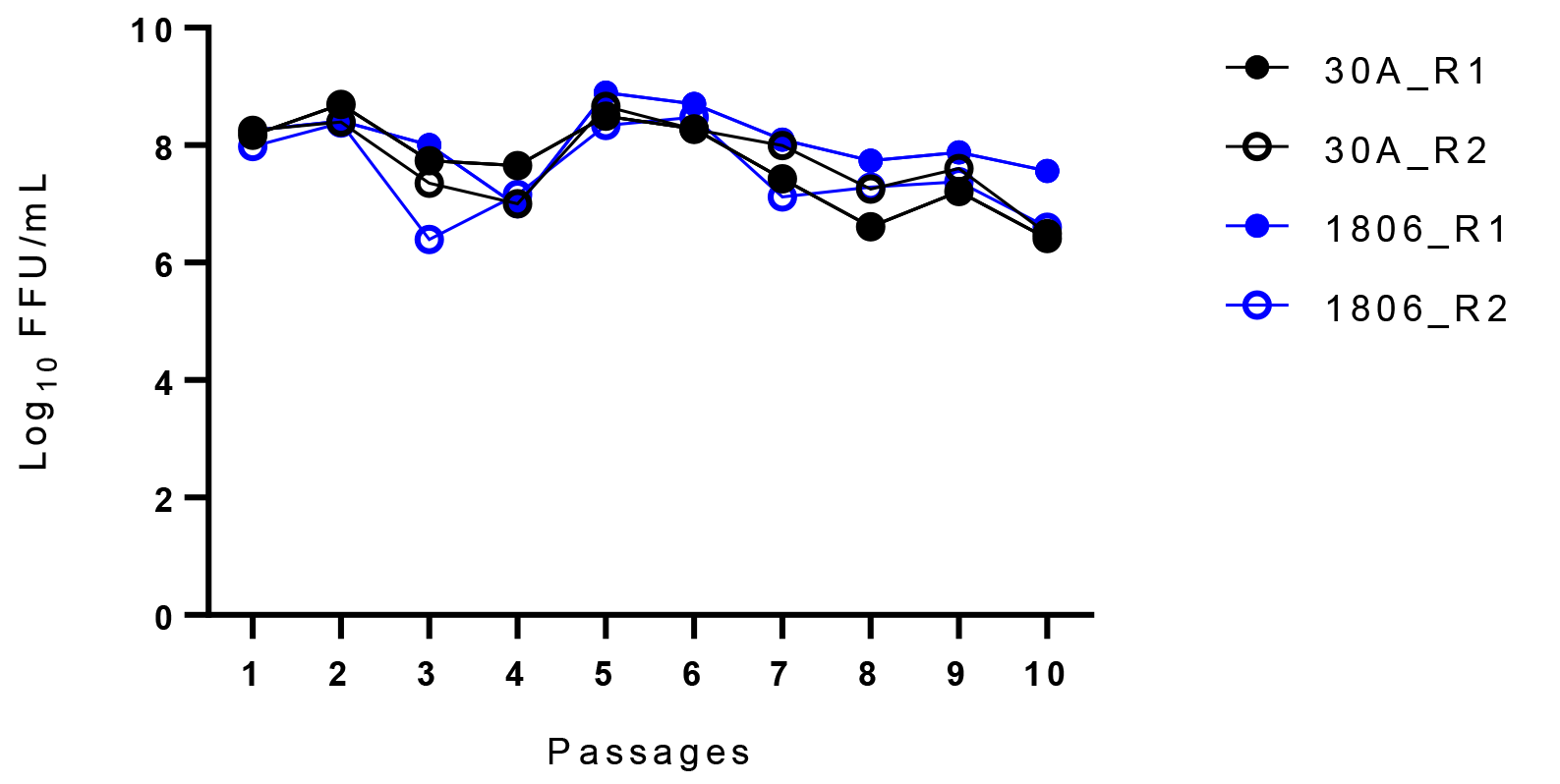


**Supplementary Fig. S2. Sequencing coverage and depth by sample.**


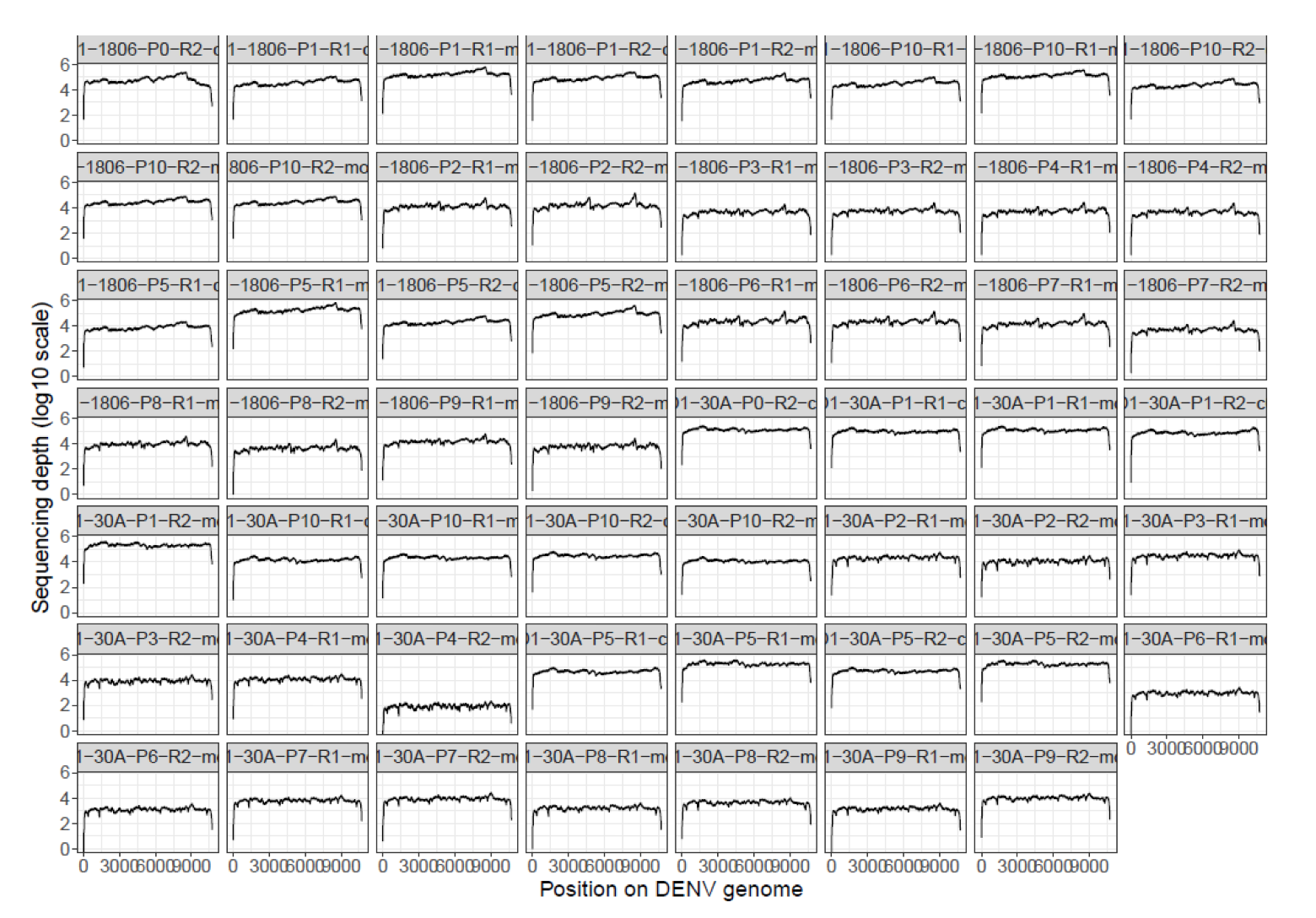


**Supplementary Fig. S3. Original Northern blots belonging to Fig. 7.**


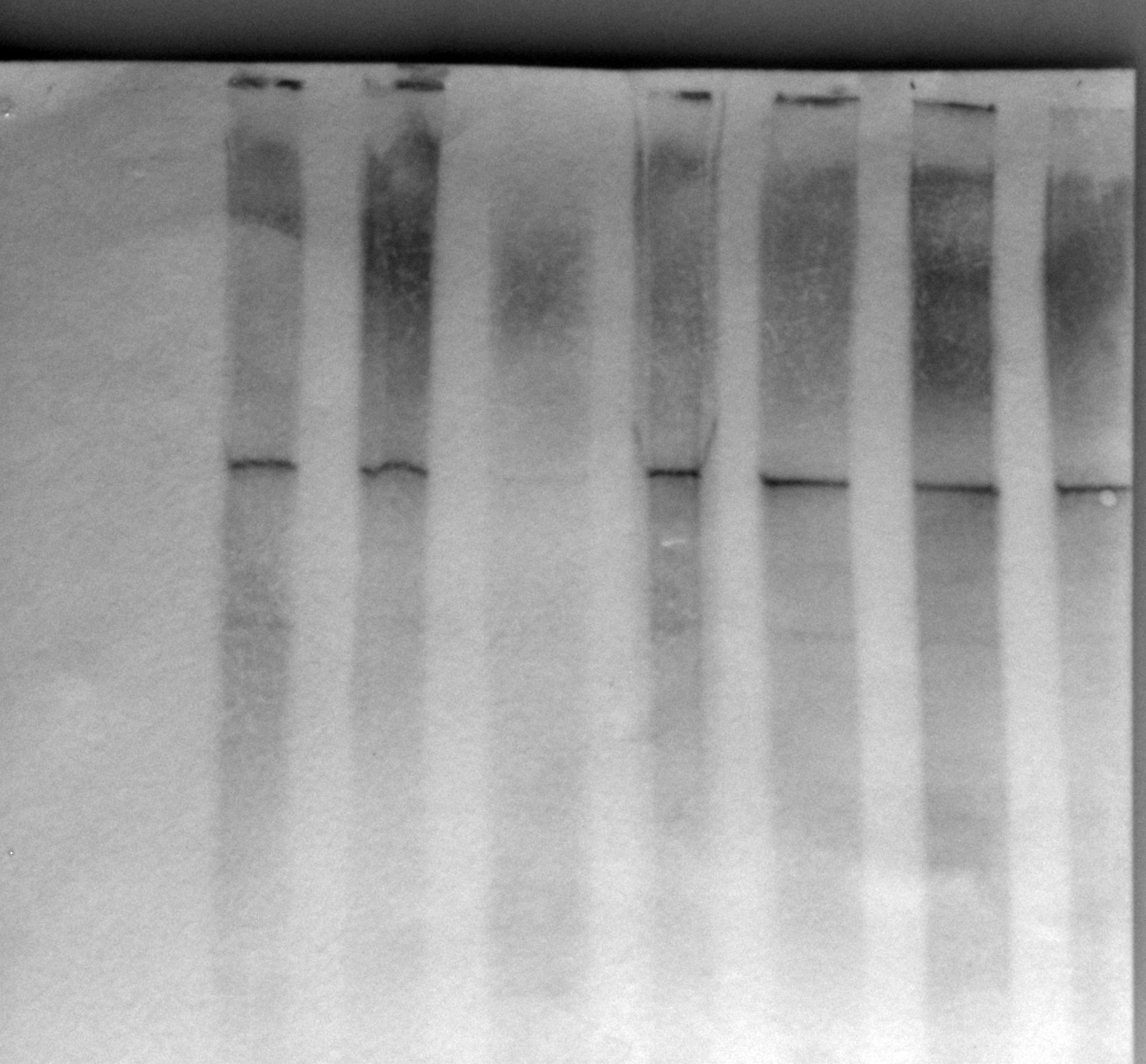


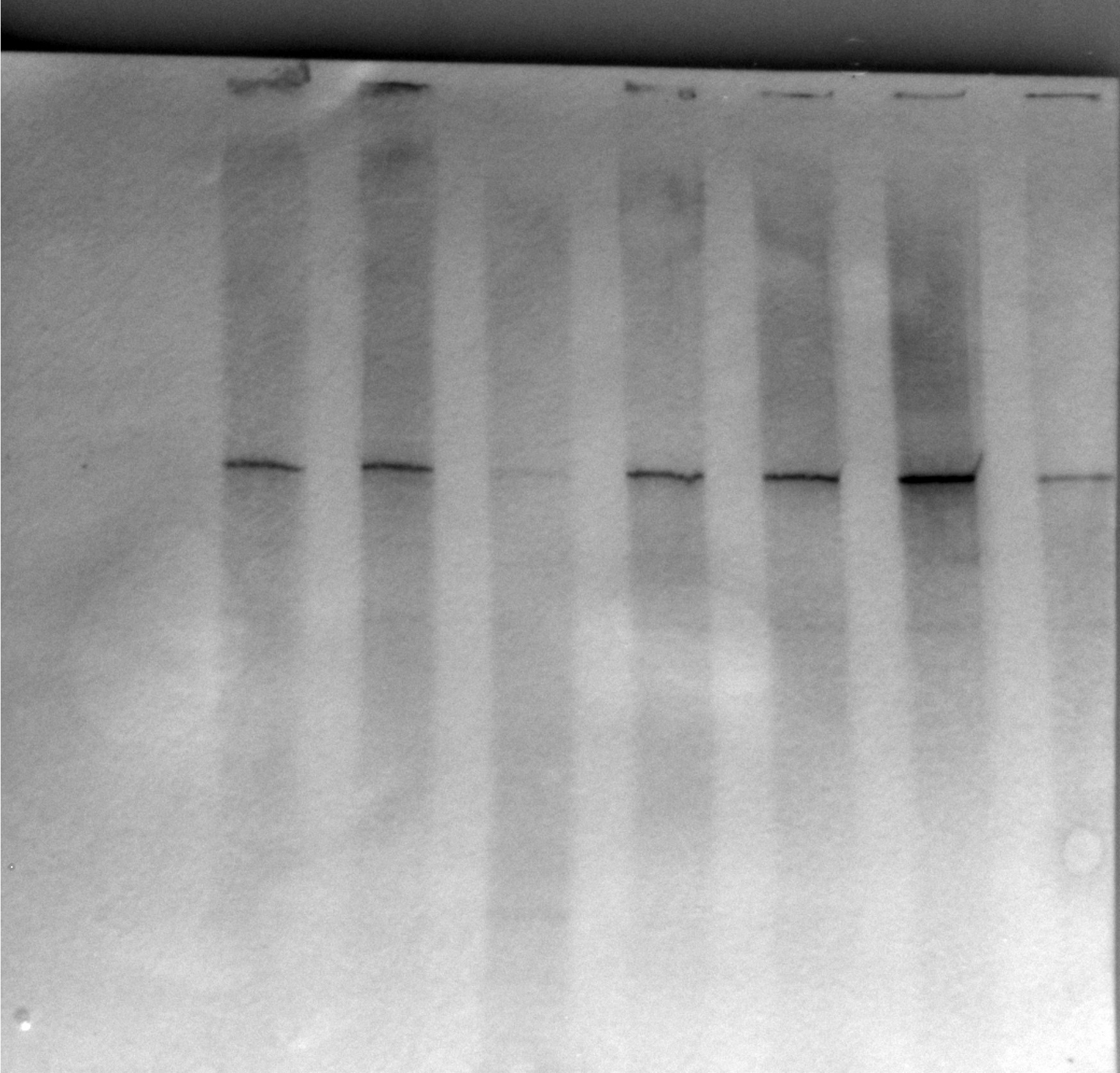


**Supplementary Table S1. Comparisons of infection rates and dissemination efficiencies between mosquitoes infected with two DENV-1 strains (1806 and 30A) and examined at different days post-infection (14 and 21).**

**Supplementary Table S2. Comparisons of infection rates and dissemination efficiencies between the 6 mosquito populations infected with a DENV-1 strain (1806 or 30A) and examined at a given day post-infection (14 or 21).**

**Supplementary Table S3. Comparisons of viral loads in mosquito heads between mosquitoes infected with two DENV-1 strains (1806 and 30A) and examined at different days post-infection (14 and 21).**

**Supplementary Table S4. Position and estimated frequency of SNVs reaching consensus level detected in the mosquito-passaged samples.**

**Supplementary Table S5. Sequences of the three reverse genetic constructs (Parental, P10 construct 1, P10 construct 2) using the deep sequencing method described in materials and methods.** Only substitutions with a mutation frequency > 5% were considered significant for further analysis. The parental strain P0 was used as the reference sequence. The two P10 constructs presented the mutation 10,418 (in grey) at a frequency close to 100% beside other mutations.

**Supplementary Table S6. Primers used to generate subgenomic DNA fragments (ISA procedure).**

**Supplementary Text S1. Sequences of the *de novo* subgenomic DNA fragments used during the ISA procedure.**

pCMV

GAATAAGGGCGACACGGAAATGTCACCCAACTGATCTTCAGCATCTTCAATATTGGCCATTAGCCATATTATTCATTGGTTATATAGCATAAATCAATATTGGCTATTGGCCATTGCATACGTTGTATCTATATCATAATATGTACATTTATATTGGCTCATGTCCAATATGACCGCCATGTTGGCATTGATTATTGACTAGTTATTAATAGTAATCAATTACGGGGTCATTAGTTCATAGCCCATATATGGAGTTCCGCGTTACATAACTTACGGTAAATGGCCCGCCTGGCTGACCGCCCAACGACCCCCGCCCATTGACGTCAATAATGACGTATGTTCCCATAGTAACGCCAATAGGGACTTTCCATTGACGTCAATGGGTGGAGTATTTACGGTAAACTGCCCACTTGGCAGTACATCAAGTGTATCATATGCCAAGTCCGCCCCCTATTGACGTCAATGACGGTAAATGGCCCGCCTGGCATTATGCCCAGTACATGACCTTACGGGACTTTCCTACTTGGCAGTACATCTACGTATTAGTCATCGCTATTACCATGGTGATGCGGTTTTGGCAGTACACCAATGGGCGTGGATAGCGGTTTGACTCACGGGGATTTCCAAGTCTCCACCCCATTGACGTCAATGGGAGTTTGTTTTGGCACCAAAATCAACGGGACTTTCCAAAATGTCGTAATAACCCCGCCCCGTTGACGCAAATGGGCGGTAGGCGTGTACGGTGGGAGGTCTATATAAGCAGAGCTCGTTTAGTGAACCG

367 last nucleotides of 3’UTR + HDR/SV40pA (WT; T)

GTGAGCCCCGTCCAAGGACGTAAAATGAAGTCAGGCCGAAAGCCACGGA**T**TGAGCAAGCCGTGCTGCCTGTGGCTCCATCGTGGGGATGTAAAAACCCGGGAGGCTGCAACCCATGGAAGCTGTACGCATGGGGTAGCAGACTAGTGGTTAGAGGAGACCCCTCCCTAGACATAACGCAGCAGCGGGGCCCAACACCAGGGGAAGCTGTACCTTGGTGGTAAGGACTAGAGGTTAGAGGAGACCCCCCGCACAACAACAAACAGCATATTGACGCTGGGAGAGACCAGAGATCCTGCTGTCTCTACAGCATCATTCCAGGCACAGAACGCCAGAAAATGGAATGGTGCTGTTGAATCAACAGGTTCTGGCCGGCATGGTCCCAGCCTCCTCGCTGGCGCCGGCTGGGCAACATTCCGAGGGGACCGTCCCCTCGGTAATGGCGAATGGGACTCGCGACAGACATGATAAGATACATTGATGAGTTTGGACAAACCACAACTAGAATGCAGTGAAAAAAATGCTTTATTTGTGAAATTAAGCGCTGGCATTGACCCTGAGGTTTACCCTCACAACGTTCCAGT

367 last nucleotides of 3’UTR + HDR/SV40pA (Mutant; C)

GTGAGCCCCGTCCAAGGACGTAAAATGAAGTCAGGCCGAAAGCCACGGA**C**TGAGCAAGCCGTGCTGCCTGTGGCTCCATCGTGGGGATGTAAAAACCCGGGAGGCTGCAACCCATGGAAGCTGTACGCATGGGGTAGCAGACTAGTGGTTAGAGGAGACCCCTCCCTAGACATAACGCAGCAGCGGGGCCCAACACCAGGGGAAGCTGTACCTTGGTGGTAAGGACTAGAGGTTAGAGGAGACCCCCCGCACAACAACAAACAGCATATTGACGCTGGGAGAGACCAGAGATCCTGCTGTCTCTACAGCATCATTCCAGGCACAGAACGCCAGAAAATGGAATGGTGCTGTTGAATCAACAGGTTCTGGCCGGCATGGTCCCAGCCTCCTCGCTGGCGCCGGCTGGGCAACATTCCGAGGGGACCGTCCCCTCGGTAATGGCGAATGGGACTCGCGACAGACATGATAAGATACATTGATGAGTTTGGACAAACCACAACTAGAATGCAGTGAAAAAAATGCTTTATTTGTGAAATTAAGCGCTGGCATTGACCCTGAGGTTTACCCTCACAACGTTCCAGT
